# Aeroplysinin-1, a Sponge-Derived Multi-Targeted Bioactive Marine Drug

**DOI:** 10.3390/md14010001

**Published:** 2015-12-22

**Authors:** Javier A. García-Vilas, Beatriz Martínez-Poveda, Ana R. Quesada, Miguel Ángel Medina

**Affiliations:** 1 Departamento de Biología Molecular y Bioquímica, Facultad de Ciencias, and IBIMA (Biomedical Research Institute of Málaga), Universidad de Málaga, Andalucía Tech, Málaga 29071, Spain; jandrovil@hotmail.com (J.A.G.-V.); bmpoveda@gmail.com (B.M.-P.); quesada@uma.es (A.R.Q.); 2 CIBER de Enfermedades Raras (CIBERER), Málaga E-29071, Spain

**Keywords:** (+)-aeroplysinin-1, sponges, marine drugs, inflammation, angiogenesis, cancer

## Abstract

Organisms lacking external defense mechanisms have developed chemical defense strategies, particularly through the production of secondary metabolites with antibiotic or repellent effects. Secondary metabolites from marine organisms have proven to be an exceptionally rich source of small molecules with pharmacological activities potentially beneficial to human health. (+)-Aeroplysinin-1 is a secondary metabolite isolated from marine sponges with a wide spectrum of bio-activities. (+)-Aeroplysinin-1 has potent antibiotic effects on Gram-positive bacteria and several dinoflagellate microalgae causing toxic blooms. In preclinical studies, (+)-aeroplysinin-1 has been shown to have promising anti-inflammatory, anti-angiogenic and anti-tumor effects. Due to its versatility, (+)-aeroplysinin-1 might have a pharmaceutical interest for the treatment of different pathologies.

## 1. Introduction: Chemical Defense of Sponges

Sponges (Phylum Poriphera), the most primitive animals, exhibit unusual features. All sponges are aquatic: over 5000 sponge species live in salty water, whereas only around 150 sponge species live in fresh water. The body of all sponges is formed of a weak aggregation of cells with mesenchymal origin inside a gelatinous matrix connected to a scaffold with calcium- or silicate carbonate and small collagen spicules. The adult animal has a sessile and filter-feeding lifestyle, and its body is very fragile since it lacks structural defenses. For this reason, a myriad of secondary metabolites is used by sponges as chemical defense mechanisms to avoid predators, other colonial organisms, and bacterial infections [1,2,3,4]. Among the secondary metabolites produced by marine sponges, a great diversity of steroids, isoprenoids, non-isoprenoids, quinones, nitrogen and nitrogen-sulfur heterocyclic compounds, alkaloids, peptides, and terpenes are included. It is worth mentioning that some of these compounds isolated from marine sponges are only synthesized in symbiotic relationships with fungi, microalgae, archea, cyanobacteria, and bacteria [5,6]. In many cases, precursors are initially synthesized and the final bio-active products are only originated after enzymatic conversion. Enzymes and lower molecular weight precursors are located in separate compartments of the sponge cells, but when the sponges are damaged, both enzymes and substrates interact, thereby yielding the bio-active secondary metabolites [7].

Although pharmaceutical industry research of natural products has declined over the past 20 years [8], traditionally the majority of new drugs translated into the clinic are natural products or compounds derived from them [9]. In particular, the pharmaceutical industry is investing in the search for novel drug candidates from marine-derived organisms showing anti-angiogenic and/or anti-tumor activities. Furthermore, other studies have been focused on identifying the biosynthetic pathways and enzymes involved in the generation of new semisynthetic or synthetic molecules [10]. Most of the drug candidates of marine origin have been isolated from marine invertebrates, marine sponges being the organisms most widely investigated. In fact, approximately 16,000 marine natural compounds have been isolated thus far from marine organisms [11], and more than 5300 of these compounds were isolated from marine sponges [12]. Some of the secondary metabolites exhibit activities that block key processes in mammalian cells, thereby avoiding disease progression. Thus, many of the bioactive compounds isolated from marine sponges have become a starting point for developing new compounds with biological activities with potential usefulness in treating different pathologies.

We devote the present work to reviewing the current state of knowledge regarding aeroplysinin-1, a multi-targeted brominated bioactive compound initially isolated from sponges belonging to the order Verongida.

## 2. Order Verongida

The order Verongida belongs to the largest class of sponges, Demosponges [13]. This order is represented by sponges that lack mineral spicules, and in which widely spaced sponging fibers forming dendritic or reticulate structures and fibers may be aggregated into bundles. Species of this order are distributed in tropical to temperate Atlantic, Mediterranean, and Pacific waters [14]. Order Verongida consists of four families (Aplysinidae, Ianthellidae, Aplysinellidae, and Pseudoceratinidae) that can be identified by taking into account the different structures and composition of their sponging fiber skeletons [15]. A common feature for members of the order Verongida is their ability to produce bromotyrosine derivates as secondary metabolites. Among these bromotyrosine metabolites, dibromoverongilquinol and aeroplysinin-1 are used by Verongida sponges as chemical weapons for their defense against other organisms [16]. These compounds are synthetized into spherolous cells, but only in response to specific symbiotic relationships with other aquatic organisms, as mentioned above [17]. In fact, several studies have reported little success in the production of secondary metabolites in sponge aquaculture because the requested symbiotic microorganisms do not grow well under the aquaculture conditions [5,18,19].

Microorganisms (mainly bacteria) that live associated with sponges may represent up to 40% of the total biomass of some sponges [20]. For instance, the estimated bacterial concentration in *Aplysina aerophoba* tissues amounts to 6.4 ± 4.6 × 10^8^ bacteria/g of sponge tissue [20]. This microbial population is highly stable in *Aplysina aerophoba*, prevailing *α*- and *γ*-Proteobacteria, Bacteroidetes, Actinobacteria, Choroflexi, and Cyanobacteria [18]. The ratio of these bacteria and their localization in the choanocyte chamber (the place where spherolous cells are located) [19] are essential for the synthesis of the characteristic brominated alkaloids of *Aplysina aerophoba* [21].

## 3. Chemical Structure and Properties of Aeroplysinin-1

Sponges belonging to the *Aplysina* genus can accumulate brominated isoxazoline alkaloids [22], such as aerophobins, isofistularin-3, aerothionin or bisoxazolidinone derivate that sometimes exceed 10% of their dry weight [7]. Long-term, quantitative seasonal variations in their production have been reported, with their maximum accumulation in summer [23]. The key biosynthetic step of these brominated alkaloids seems to be their bromination catalyzed by a flavin-dependent halogenase [13]. These compounds become substrates for their enzymatic bioconversion into aeroplysinin-1 when sponge tissues are damaged by predators [24,25]. This biotransformation was initially discovered by Teeyapant and Proksch [26]. Aeroplysinin-1 can be later converted into the dienone amide verngiaquinol by a nitrile hydratase that has been isolated, partially purified and characterized from *Aplysinia cavernicola* [27].

**Figure 1:**
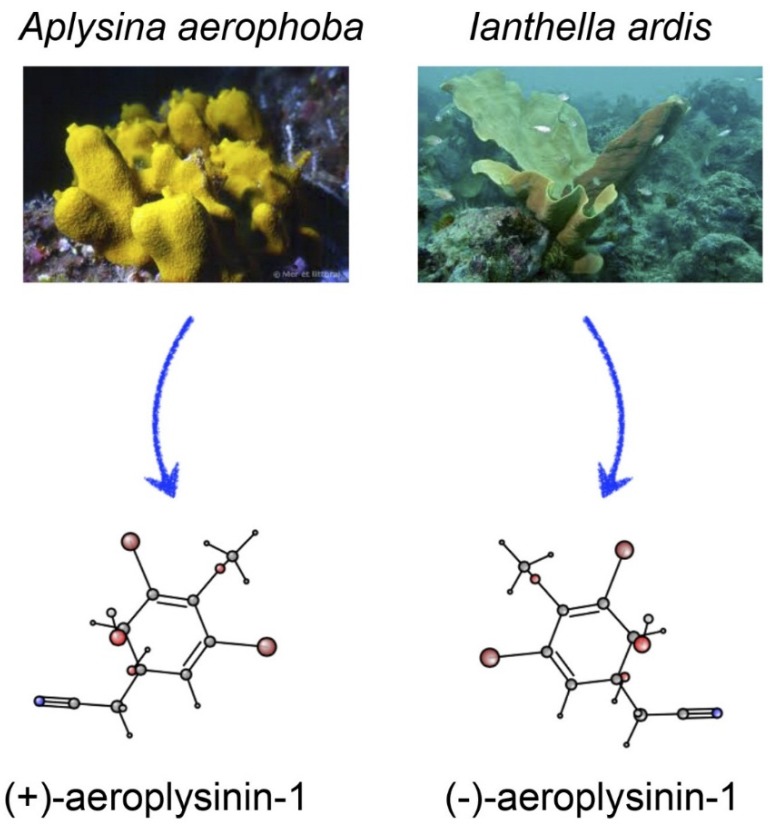
Natural sources and chemical structures of (+)- and (−)-aeroplysinin-1.

Aeroplysinin-1 is a chiral, optically active molecule (see Figure 1). Regarding many other drugs, only one of their enantiomers is responsible for their bioactivities. The levorotatory enantiomer ((−)-aeroplysinin-1) was isolated from *Ianthella ardis* by Fulmor and co-workers [28]. (+)-Aeroplysinin-1 was the first brominated derivate from *Aplysina aerophoba* isolated by Fattorusso [29]. In 1975, the chemical synthesis of aeroplysinin-1 was achieved [30]. Although both optical isomers exhibited very similar antibacterial activity *in vitro*, only the dextrorotatory enantiomer has been further investigated. Through theoretic Raman optical activity (ROA) spectra, an alternative method for the configurational assignment has been proposed. Furthermore, through a combination of theoretical and experimental chiroptical studies, the most stable solution conformations have been determined [31]. This study elucidated that the most stable conformer of (+)-aeroplysinin-1 is the 1*S*,6*R* stereoisomer. Its complete IUPAC systemic name is 2-((1*S*,6*R*)-3,5-dibromo-1,6-dihydroxy-4-methoxycyclohexa-2,4-dien-1-yl)-acetonitrile. (+)-Aeroplysinin-1 has a rigid skeleton due to the *cis*-diene and four flexible groups capable of interacting with its biological targets [31].

## 4. Reported Antibiotic, Antimicrobial and Antiviral Properties of Aeroplysinin-1 as a Defense Metabolite

Aeroplysinin-1 shows antibiotic, algicidal and gastropod-repellent activities, in all cases in the micromolar range. The agar diffusion assay shows that aeroplysinin-1 has relevant growth inhibition potency against *Bacillus cereus*, *Bacillus subtilis*, *Staphylococcus aureus*, *Staphylococcus albus*, *Vibrio anguillarum*, *Flexibacter* sp. *and Moraxella* sp. In contrast, (+)-aeroplysinin-1 did not show any effect on Gram-negative bacteria, such as *Pseudomonas aeruginosa*, or yeasts, including *Saccharomyces cerevisiae* [29,32,33]. Since some of the bacteria whose growth is inhibited by (+)-aeroplysinin-1 are pathogenic for humans, this compound has an antibiotic potential for treating human infections that deserves to be further evaluated. Furthermore, (+)-aeroplysinin-1 is able to inhibit (in a dose-response manner) the growth of the marine microalgae *Coscinodiscus wailesii* and *Prorocentrum minimum*. This is a very interesting effect, since both microalgae species are dinoflagellates causing harmful blooms. (+)-Aeroplysinin-1 has also been shown to exert a repellent activity in a time-dependent increase and dose-response manner against marine snails (*Littorina littorea*) [32].

(+)-Aeroplysinin-1 also exhibits an antiviral activity toward HIV-1 caused by inhibition of its reverse transcriptase activity. In fact, (+)-aeroplysinin-1 blocks the RNA-dependent DNA polymerase activity and the RNase H activity in a dose-dependent manner, with a high percentage of inhibition (74%) at 20 μM. Moreover, 10 μM (+)-aeroplysinin-1 inhibits the nuclear import of both the virus reverse transcriptase and retroviral DNA by 67% [34]. These data indicate that (+)-aeroplysinin-1 is able to inhibit key stages of the HIV-1 infection.

**Figure 2:**
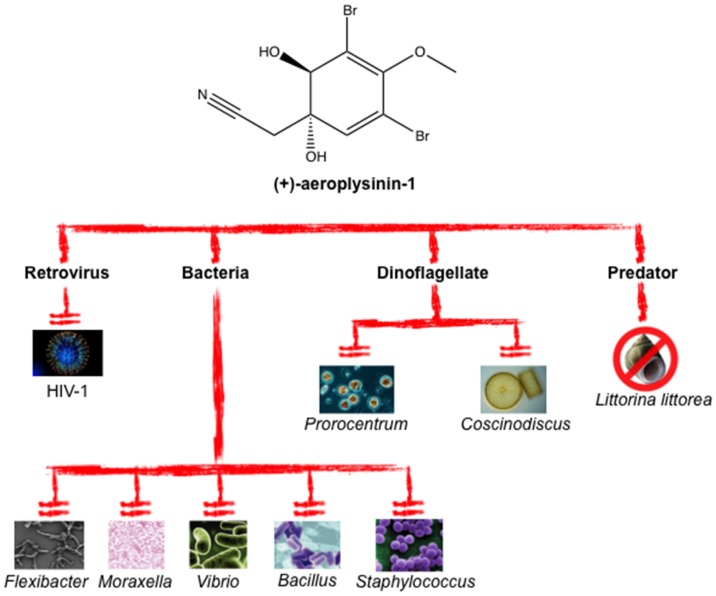
(+)-Aeroplysinin-1 exhibits a wide spectrum of antibiotic action.

Table 1 shows the IC_50_ values reported for several microorganisms treated with (+)-aeroplysinin-1. Figure 2 summarizes the antibiotic properties reported for (+)-aeroplysinin-1.

**Table 1:** IC_50_ values of the antibiotic action demonstrated for (+)-aeroplysinin-1 on bacteria, microalgae and viruses.

Microorganism Species	IC_50_ (μM)	Reference
*P. phosphoreum*	3.5	[32]
*C. wailesii*	5.6	[32]
*P. minimum*	7.0	[32]
HIV-1	14.6	[34]

IC_50_ is the drug concentration causing 50% survival inhibition in endothelial cell lines.

## 5. Aeroplysinin-1 as an Anti-Inflammatory Compound

The inflammatory response is a defense mechanism that protects against pathogenic organisms [35]. However, it is also related to allergy, pathological angiogenesis and tumor progression [36,37]. Once the inflammatory response is triggered, endothelial cells that are close to the inflammation focus release new inflammatory molecules and subsequently contributing to a positive feedback loop [38].

*In vitro* assays with human umbilical vein endothelial cells (HUVEC) and human monocytic leukemia cell line (THP-1) revealed that (+)-aeroplysinin-1 treatment was able to modulate key proteins of the inflammatory process [38]. In the case of HUVEC, treatments of HUVEC with 10–20 µM (+)-aeroplysinin-1 decreased the expression levels of mRNA and the protein levels corresponding to the inflammatory mediators monocyte chemoattractant protein 1 (MCP-1), thrombospondine-1 (TSP-1), and cyclooxygenase 2 (COX-2). These treatments also decreased the protein levels of interleukin 1 alpha (Il-1α) and matrix metalloproteinase 1 (MMP-1), as summarized in Table 2. On the other hand, THP-1 cells treated with 10 µM (+)-aeroplysinin-1 showed decreased expression levels of MCP-1 and COX-2, as determined by real-time qPCR [38]. Taken together, these results strongly suggest that (+)-aeroplysinin-1 could be a novel anti-inflammatory compound with potential pharmacological action on inflammation-dependent diseases.

**Table 2:** Molecular targets that are modulated by (+)-aeroplysinin-1 in endothelial cells.

Cell Line	Treatment (μM)	Target	Effect	Activity	Reference
EVLC-2	2.5	MMP-2	Decrease	Anti-angiogenic	[38]
HUVEC	2.5	MMP-2	Decrease	Anti-angiogenic	[38]
HUVEC	10	MCP-1	Decrease	Anti-inflammatory	[38]
HUVEC	10	TSP-1	Decrease	Anti-inflammatory	[38]
HUVEC	10	COX-2	Decrease	Anti-inflammatory	[38]
HUVEC	20	Il-1α	Decrease	Anti-inflammatory	[38]
HUVEC	20	MMP-1	Decrease	Anti-inflammatory	[38]
RF-24	2.5	MMP-2	Decrease	Anti-angiogenic	[38]
BAEC	10	Cleaved lamin-A	Increase	Apoptogenic	[39]
BAEC	10	Caspase-2, -3, -8, -9	Increase	Apoptogenic	[39]
BAEC	10	Cytochrome C	Increase in cytoplasm	Apoptogenic	[39]
HUVEC	10	p-Bad	Increase	Apoptogenic	[39]
BAEC	3	MMP-2	Decrease	Anti-angiogenic	[40]
BAEC	3	PA	Decrease	Anti-angiogenic	[40]
BAEC	3	PAI	Increase	Anti-angiogenic	[40]

BAEC: bovine aortic endothelial cells, HUVEC: human umbilical vein endothelial cells, EVLC-2: SV40 large T-antigen immortalized human umbilical vein cells; RF-24: papillomavirus 16 E6/E7 immortalized human umbilical vein cells; HMEC: human microvascular endothelial cells.

## 6. Aeroplysinin-1 as an Anti-Angiogenic Compound

The proper functioning and modulation of the angiogenesis process is essential during embryonic development. In adults, angiogenesis is tightly restricted and deregulated. Persistent angiogenesis is related to the progression of a number of so-called angiogenesis-dependent diseases, including retinopathies, arthritis, psoriasis and cancer, among many others [41]. Endothelial cells are the main ones responsible for the formation of new capillaries in angiogenesis. (+)-Aeroplysinin-1 inhibits the growth of endothelial cells (Table 3). This effect can be due, at least in part, to the apoptogenic effects of this compound [39].

**Table 3:** IC_50_ values of (+)-aeroplysinin-1 on endothelial cell proliferation.

Endothelial Cell Line	IC_50_ (μM)	Reference
HUVEC	4.7	[38]
EVLC-2	3.0	[38]
RF-24	2.8	[38]
HMEC	2.6	[38]
BAEC	2.1	[40]

BAEC: bovine aortic endothelial cells, HUVEC: human umbilical vein endothelial cells, EVLC-2: SV40 large T-antigen immortalized human umbilical vein cells; RF-24: papillomavirus 16 E6/E7 immortalized human umbilical vein cells; HMEC: human microvascular endothelial cells.

In bovine aortic endothelial cells (BAEC), (+)-aeroplysinin-1 induced apoptosis with nuclear condensation and fragmentation. Up to a six-fold increase of the sub G1 population was observed when the treated BAEC cell cycle was analyzed by flow cytometry after iodide propidium staining. This evidence was confirmed with the use of the 7-AAD/Annexin V assay [39]. This analysis demonstrated that (+)-aeroplysinin-1 induced late apoptosis. Moreover, the apoptogenic effect of the compound was elucidated, showing that it was induced through the intrinsic apoptotic pathway, as revealed by the release of cytochrome C and the increase of caspase (-2, -3, -8, and -9) activities [39]. Along with these dramatic effects on endothelial cell proliferation and survival, (+)-aeroplysinin-1 has been shown to inhibit some other key steps of the angiogenic process, as revealed by an array of *in vitro* assays, such as the tube formation on Matrigel, and both the migration and chemoinvasion assays in a modified Boyden chamber. Both endothelial migration and invasion are mediated by extracellular matrix remodeling proteases, such as MMP-2 and urokinase-type plasminogen activator (uPA). Interestingly, (+)-aeroplysinin-1 treatment of endothelial cells decreased their levels of MMP-2 and uPA, whereas it increased plasminogen activator inhibitor (PAI) levels [40]. The *in vitro* anti-angiogenic effects of (+)-aeroplysinin-1 was also demonstrated in human immortalized endothelial cells, both macrovascular (EVL-2 and RF-24) and microvascular (HMEC) [38]. (+)-Aeroplysinin-1 inhibited the formation of “tubule-like” structures by any of these cells lines grown on Matrigel. For the microvascular endothelial cells, the concentration of (+)-aeroplysinin-1 required to completely inhibit the tubule-like structure formation on Matrigel was lower than those required in the case of macrovascular cells. These three types of immortalized endothelial cells also decreased their MMP-2 levels when treated with (+)-aeroplysinin-1 (Table 2).

The results obtained with the *in vivo* chick chorioallantoic membrane (CAM) assay showed that CAM treatment with (+)-aeroplysinin-1 produced a devastating effect on the developing vessels and severe disorganization of the pre-existing vessels. These effects were probably due to the induction of apoptosis in vascular cells and their progenitors, as demonstrated by the TUNEL assay in the quail CAM [40]. Moreover, (+)-aeroplysinin-1 provoked the inhibition of the bFGF-mediated cell invasion in the *in vivo* Matrigel plug assay.

All these results demonstrate that (+)-aeroplysinin-1 is a potent inhibitor of both *in vitro* and *in vivo* angiogenesis. An independent research group has described the synthesis of several derivatives of (+)-aeroplysinin-1. Two of them, namely the epoxy ketone and the azolactone derivatives, were shown to have much stronger anti-angiogenic effects than (+)-aeroplysinin-1 in the tubule formation on Matrigel assay [42].

## 7. Aeroplysinin-1 as an Anti-Tumor Compound

In the last decades, one of the challenges of cancer research has been the discovery of new molecular targets involved in neoplastic diseases, and new active drugs to treat them. Several reports have evaluated the (+)-aeroplysinin-1 activity in different tumor cell lines and its effects on specific molecular targets of tumors. (+)-Aeroplysinin-1 shows cytostatic [43] and cytotoxic effects (Table 4) on different types of tumor cells lines.

**Table 4:** IC_50_ values of the anti-proliferative effect demonstrated for (+)-aeroplysinin-1 on different tumor cells.

Tumor cell line	IC_50_ (μM)	Reference
HT-1080	2.3	40
HTC-116	4.7	40
HeLa	3.0	43
THP-1	10.0	45
NOMO-1	17.0	45
HL-60	5.0	45

HeLa: human cervix carcinoma, THP-1: human acute monocyte cell line, NOMO-1: human acute myeloid leukemia, HL-60: human promyelocytic leukemia cells, HTC-116: colorectal carcinoma cell line, HT-1080: human fibrosarcoma cell line.

For two human estrogen-responsive breast cancer cell lines (namely, MCF-7 and ZR-75-1 cells), it has been shown that (+)-aeroplysinin-1 blocks their EGF-dependent proliferation, claiming that this observed effect was caused by an inhibitory effect of (+)-aeroplysinin-1 on EGFR phosphorylation [44].

Very recently, a potent cytotoxic effect of (+)-aeroplysinin-1 on both acute myeloid cells (NOMO-1) and acute monocytic cells (THP-1) has been reported [45]. In this work, (+)-aeroplysinin-1 treatment was shown to be able to stimulate the phosphorylation of histone H2AX (γ-H2AX), which is a marker of the DNA damage. Furthermore, the same treatment also decreased the histone 3 level of phosphorylation, whereas it increased the cleavage of caspase 3, PARP, p16, and p21. These proteins are involved in mitosis, death cellular pathway, and cyclin-dependent kinase inhibition. These results could explain the increase of the sub-G1 phase population and the decrease of S phase population observed in an analysis of cell cycle.

## 8. Is (+)-Aeroplysinin-1 a Receptor Tyrosine Kinase Inhibitor?

Many external ligands induce specific cellular responses through their binding to membrane cell receptors. The dysregulation of receptor tyrosine kinases is a common feature for several types of cancer and for this reason receptor tyrosine kinases have become molecular targets for cancer treatment [46]. One of these molecular targets for cancer treatment is EGFR, the specific receptor for epidermal growth factor. As mentioned above, it has been claimed that (+)-aeroplysinin-1 inhibits EGFR phosphorylation [44]. In fact, the title of that scientific article claimed that (+)-aeroplysinin-1 would inhibit the intrinsic protein tyrosine kinase activity of the EGF-receptor kinase complex from human breast cancer cells. This claim has been mentioned and referred to since 1990 by others [38,40,47,48,49] and can also be found in the accompanying information provided by the (+)-aeroplysinin-1 vendor *Santa Cruz Biotechnology* on its web page [50]. However, this claimed inhibitory effect of (+)-aeroplysinin-1 on EGFR phosphorylation has been questioned by other groups [47,51]. Initially accepting that (+)-aeroplysinin-1 had been shown [44] to inhibit EGFR tyrosine kinase in an *in vitro* test system, Hinterding *et al.* published an article in 1998 describing the synthesis and biological evaluation of (+)-aeroplysinin-1 analogs as a new class of receptor tyrosine kinase inhibitors [47]. Data contained in that article and presented in its Table 1 indeed shows that four out of the twelve tested (+)-aeroplysinin-1 analogs inhibited EGFR tyrosine kinase activity with IC_50_ values within the micromolar range of concentration. However, this very same table declares (+)-aeroplysinin-1 to be "inactive," that is, unable to inhibit EGFR kinase activity within the micromolar range of concentration. Our group very recently has reported the result of an *in vitro* kinase inhibition screening, showing that 20 µM (+)-aeroplysinin-1 was unable to produce any significant inhibitory effect on 25 different protein kinases, including receptor tyrosine kinases like FGFR1-4, VEGFR1-3, TGFR1-2, and c-Met [51]. Among the intracellular protein kinases tested, neither Akt nor ERK were inhibited by (+)-aeroplysinin-1. Akt and ERK are key effectors of the signaling pathways controlling cell proliferation and survival [52]. Notwithstanding this lack of direct *in vitro* inhibitory effect of (+)-aeroplysinin-1 on these protein kinases, we could demonstrate that in both BAEC and HUVEC endothelial cells (but not in HCT-116 and HT-1080 tumor cells and in mouse embryonic fibroblasts) 2 h of treatment with 20 μM (+)-aeroplysinin-1 was enough to induce a strong decrease in the phosphorylation levels of Akt and ERK [51].

A docking study recently reported predicts that (+)-aeroplysinin-1 could interact with the catalytic kinase domain of EGFR and suggests that a similar kind of interaction could give account of the potential inhibitory effects on (+)-aeroplysinin-1 on other receptor tyrosine kinases such as VEGFR2 [53]. The predictions of this computational study do not seem to be valid in the real world, taking into account the previously mentioned experimental results that have demonstrated that (+)-aeroplysinin-1 is unable to inhibit kinase activities.

## 9. Conclusion: Potential and Perspectives of (+)-Aeroplysinin-1 as a Novel Multi-Targeted Drug

**Figure 3:**
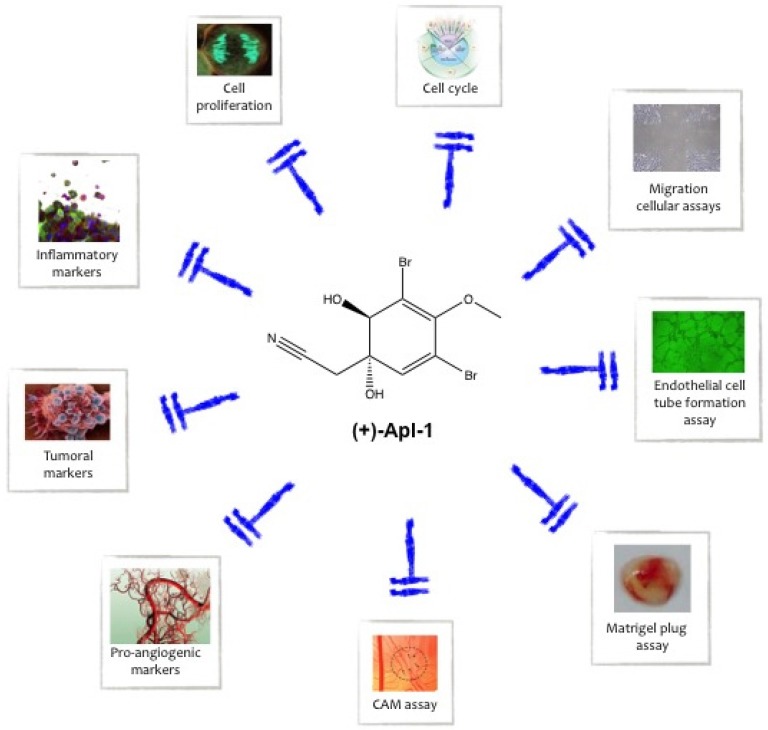
Summary of the biological processes inhibited by (+)-aeroplysinin-1 in animal and human cells and tissues.

(+)-Aeroplysinin-1 is a brominated alkaloid compound produced by Verongida sponges as a chemical weapon to protect themselves from pathogens and predators. Similar to many other marine drugs, it has a wide range of potential pharmacological uses due to its effects on very different targets. (+)-Aeroplysinin-1 has anti-inflammatory, anti-angiogenic, and anti-proliferative effects and it can compromise cell survival by inducing apoptosis (see Figure 3).

Uncontrolled proliferation caused by limitless replicative potential, cell sufficiency in growth signal and/or insensitivity to growth inhibitory signals, evading apoptosis, inflammation and sustained angiogenesis are some of the so-called *hallmarks of cancer*, an integrative concept successfully introduced by Hanahan and Weinberg [37]. Since all the hallmarks of cancer are potential therapeutic targets to treat and inhibit cancer, the search for new molecules capable of inhibiting any of them is a very active research field.

In the last few years, marine species have been used as a prolific source of molecular diversity with many new bioactive compounds identified for pharmaceutical use [54,55]. Our group has actively contributed to the search and characterization of new modulators of angiogenesis (and inhibitors of some key hallmarks of cancer) of marine origin [56,57,58,59]. In particular, we have contributed to bringing to a close the debate on the claimed receptor tyrosine kinase inhibitory effect of (+)-aeroplysinin-1 and to identify the hallmarks of cancer mentioned previously as neoplasia features targeted by this compound [38,39,40,51]. However, the list of currently accepted hallmarks of cancer also includes genome instability and mutation accumulation, metabolic reprogramming, avoiding immune destruction and activation of tissue invasion and metastasis [37]. The interest to demonstrate whether (+)-aeroplysinin-1 could target any of these other hallmarks of cancer warrants future research in this direction.

On the other hand, inflammation and angiogenesis are not only linked to cancer. It is accepted that inflammation is associated with the etiology of many pathological conditions and that there are a number of diseases dependent on angiogenesis. Additional experimental efforts will be needed in the future to explore the potential use of (+)-aeroplysinin-1 and derivatives for the treatment of non-neoplastic inflammatory and angiogenic diseases. Finally, as a promising, novel multi-targeted drug, (+)-aeroplysinin-1 should enter into translational research programs.
